# Nanocasting synthesis of BiFeO_3_ nanoparticles with enhanced visible-light photocatalytic activity

**DOI:** 10.3762/bjnano.11.164

**Published:** 2020-12-07

**Authors:** Thomas Cadenbach, Maria J Benitez, A Lucia Morales, Cesar Costa Vera, Luis Lascano, Francisco Quiroz, Alexis Debut, Karla Vizuete

**Affiliations:** 1 Universidad San Francisco de Quito, Colegio de Ciencias e Ingenierias, El Politécnico, Diego de Robles y Vía Interoceánica, 170901, Quito, Ecuador; 2 Departamento de Física, Facultad de Ciencias, Escuela Politécnica Nacional, Ladrón de Guevara E11-253, Quito 170517, Ecuador; 3 Departamento de Ciencia de los Alimentos y Biotecnología DECAB, Escuela Politécnica Nacional, Ladrón de Guevara E11-253, Quito 170517, Ecuador; 4 Centro de Nanociencia y Nanotecnología, Universidad de las Fuerzas Armadas ESPE, Av. Gral. Rumiñahui s/n, Sangolquí, PO Box 171-5-231B, Ecuador

**Keywords:** bismuth ferrite (BiFeO_3_), dye, nanocasting, nanoparticles, photocatalysis, rhodamine B, SBA-15

## Abstract

In this work, monodisperse BiFeO_3_ nanoparticles with a particle diameter of 5.5 nm were synthesized by a nanocasting technique using mesoporous silica SBA-15 as a hard template and pre-fabricated metal carboxylates as metal precursors. To the best of our knowledge, the synthesized particles are the smallest BiFeO_3_ particles ever prepared by any method. The samples were characterized by X-ray powder diffraction, transmission electron microscopy and UV–vis diffuse reflectance spectroscopy. The phase purity of the product depends on the type of carboxylic acid used in the synthesis of the metal precursors, the type of solvent in the wet impregnation process, and the calcination procedure. By using tartaric acid in the synthesis of the metal precursors, acidified 2-methoxyethanol in the wet impregnation process and a calcination procedure with intermediate plateaus, monodisperse 5.5 nm BiFeO_3_ nanoparticles were successfully obtained. Furthermore, the nanoparticles were applied in photodegradation reactions of rhodamine B in aqueous solution under visible-light irradiation. Notably, the cast BiFeO_3_ nanoparticles demonstrated very high efficiencies and stability under visible-light irradiation, much higher than those of BiFeO_3_ nanoparticles synthesized by other synthetic methods. The possible mechanism in the photodegradation process has been deeply discussed on the basis of radical trapping experiments.

## Introduction

In the face of a continuously growing demand, the production of safe and readily available water is one of the biggest challenges humanity is facing currently. Studies suggest that by 2025, half of the world’s population will be living in water-stressed areas [1–2]. Consequently, water-related crises are rising around the globe due to population growth, climate change, and environmental damage, making the scenario worse than ever, especially in low- and middle-income countries. In particular, water pollution is one of the most challenging problems to face [2–4]. The quantity of wastewater produced as well as the overall pollution level are continuously increasing around the globe [5–6]. For instance, more than 80% of the globally generated wastewater flows back into the environment untreated. One of the most polluting industries is the textile industry, in which vast quantities of toxic and harmful organic and inorganic chemicals are utilized [7]. The effluents resulting from these processes contain residues of very stable and toxic dyes such as rhodamine B (RhB), methyl orange, or methylene blue [8]. Due to the increasing demand for safe water, wastewater itself, coming from all types of industries including the textile industry, is considered to be a reliable alternative source of water. Thus, it is becoming part of the solution to the water problem that we face today [5]. Regarding this, the removal of organic dyes from industrial wastewater is absolutely essential and, consequently, it has become a focus of attention of the scientific community over the past two decades. A number of techniques have been reported for the removal of dyes from wastewater such as precipitation (chemical coagulation, flocculation), membrane and electrochemical processes, as well as biological treatment methods [9]. The main disadvantages of these treatment methods are very often incomplete dye removal, high energy consumption and capital cost, and the production of secondary waste products that require further treatment. Advanced oxidation processes, in general, and heterogeneous semiconductor photocatalysis, in particular, are promising candidates to efficiently treat wastewater as they are cost-effective and green treatment methods in which the complete mineralization of dyes is achieved by the generation of hydroxyl and superoxide anion radicals [10–11]. Traditional photocatalysts, such as TiO_2_ or ZnO, provide chemical stability and facile preparation methods [12–13]. However, their environmental benefit in large-scale industrial applications has been limited due to their relatively large bandgaps along with their susceptibility to the fast recombination of photogenerated electron–hole pairs, leading to inefficient photocatalytic activity under visible-light or solar irradiation [14–18]. Thus, the development of photocatalysts with narrow bandgaps in the visible-light region in combination with a slow electron–hole recombination has attracted a great deal of interest [18].

Bismuth ferrite (BiFeO_3_) is one of those photocatalysts and has been intensively researched in the past few years due to its narrow bandgap in the visible-light region (2.1–2.8 eV), high chemical stability, and ferroelectric and ferromagnetic properties at room temperature [19–27]. Furthermore, slow electron–hole recombination as a result of efficient separation of the charge carriers has yielded an enhancement of its photocatalytic activity [20]. Since the photocatalytic degradation of organic molecules using a metal oxide photocatalyst is a heterogeneous process, it is obvious that efficiency and overall catalytic performance are strongly correlated to the number of active sites on the catalyst surface area and, thus, to the particle size. The smaller the particle, the greater the specific surface area is and, thus, the more active sites are available for photodegradation [15,18]. In addition, it was shown that size and shape of BiFeO_3_ particles have a direct influence on the bandgap of the material. Smaller BiFeO_3_ particles tend to have lower bandgaps than larger particles [28–30]. The synthesis of pure single-phase BiFeO_3_ is very challenging, due to the volatile character of bismuth at temperatures higher than 400 °C, which leads to the formation of undesired phases such as α-Fe_2_O_3_, α- and β-Bi_2_O_3_ or Bi_2_Fe_4_O_9_ [24,31–33]. In some cases, secondary phases can be removed by further treatment such as leaching with nitric acid. However, acid leaching as well as other purification steps most often lead to lower yields and to the formation of larger particles. A promising synthetic technique for the synthesis of pure-phase BiFeO_3_ is the in situ formation of Fe/Bi mixed metal complexes by using different complexing agents such as carboxylic acids. During the calcination process the carboxylate ligands in the Fe/Bi complexes decompose to CO_2_ and BiFeO_3_ is formed [29]. This synthetic technique requires calcination temperatures of at least 500 °C for a minimum of 1–2 h, which can lead to particle growth, agglomeration and to the formation of secondary phases. It should be noted that, in general, low calcination temperatures favor the generation of smaller particles with narrow bandgaps and promote the formation of pure-phase BiFeO_3_ materials [19,22–24,34–35].

A proposed solution to control particle growth and to guarantee the formation of pure phases is the use of a nanocasting technique [36–38]. Here, previously synthesized mesoporous matrices act as sacrificial rigid molds, also known as hard templates. The synthesis of the desired material takes place in the pores of the template, which serves as a nanoreactor for the reaction. Thus, particle growth is restricted to the pore size of the porous matrix. After removal of the mold, the precursors are characterized by a maximum particle diameter corresponding to the pore size of the porous matrix and, consequently, by a high specific surface area. Silica matrices, such as Santa Barbara Amorphous silica (SBA-15) or Korean Advanced Institute of Science and Technology silica (KIT-6), have been used successfully as hard templates to synthesize metal oxide particles and, in particular, nanometer-sized ferrite particles due to their availability, low cost and relative inertness [36–41].

In the present work, we show the synthesis of pure-phase BiFeO_3_ nanoparticles with a particle diameter of 5.5 ± 1.0 nm by a wet impregnation nanocasting technique using SBA-15 as the hard-template and in situ generated mixed metal carboxylates as precursors. Reaction optimization included the study of the influence of different organic ligands, solvents, molar ratio of the precursors, and calcination temperature. The techniques used to analyze the BiFeO_3_ nanomaterial are powder X-ray diffraction (XRD), transmission electron microscopy (TEM) and UV–visible reflectance spectroscopy. Furthermore, we investigated the photocatalytic efficiency of this nanomaterial under visible light in the degradation of rhodamine B (RhB) as a model pollutant under various conditions.

## Experimental

### Synthesis of BiFeO_3_ nanoparticles

To successfully synthesize pure-phase BiFeO_3_ nanoparticles inside the pores of SBA-15, we first studied the influence of different organic acids, solvents, bismuth content, and calcination procedures on the synthesis of BiFeO_3_.

Bismuth nitrate pentahydrate Bi(NO_3_)_3_·5H_2_O, ferric nitrate nonahydrate Fe(NO_3_)_3_·9H_2_O, concentrated nitric acid (HNO_3_, 69%), triblock copolymer poly(ethylene glycol)-*block*-poly(propylene glycol)-*block*-poly(ethylene glycol) (Pluronic P123, *M*_av_ = 5800, EO_20_PO_70_EO_20_), tetraethylorthosilicate (TEOS), ethanol, hydrochloric acid (HCl, 37%), and 2-methoxyethanol were purchased from Sigma-Aldrich and used without any further purification except for the last. We observed slightly better yields of BiFeO_3_ with no parasitic phases, such as Bi_2_O_3_, with freshly distilled 2-methoxyethanol dried over a molecular sieve.

Mesoporous silica SBA-15 with a pore size diameter of approximately 8 nm was prepared according to the procedures describe elsewhere [38,42]. Please find the corresponding small-angle xrd pattern (Figure S3), BET analysis (Figure S4, Table S2) and a TEM image (Figure S5) of the synthesized SBA-15 in Supporting Information File 1.

The influence of different complexing agents was investigated using slightly acidified H_2_O as a solvent. In a typical experiment, 1.010 g (2.5 mmol) of Fe(NO_3_)_3_·9H_2_O, 1.249 g (2.58 mmol) of Bi(NO_3_)_3_·5H_2_O, and 2.5 mmol of the respective organic acid were dissolved in 30 mL of HNO_3_-acidified water at pH 3. After stirring the solution for 4 h at room temperature the solvent was removed by evaporation using a membrane pump vacuum on a rotary evaporator at 75 °C. Then, the sample was dried for 16 h in a ventilated oven at 75 °C until a dry powder was obtained. Finally, the sample was calcined at 500 °C for 1 h with two intermediate plateaus at 200 °C and 250 °C for 2 h each (see pathway 1, Table S1 in Supporting Information File 1). As a reference, BiFeO_3_ was synthesized as above without the addition of any organic acid.

The effect of the different solvents was studied using acidified H_2_O (see above), ethanol, and 2-methoxyethanol in the synthesis of BiFeO_3_. The experiments were performed as described above with tartaric acid as a complexing reagent but with acidified ethanol (three drops of conc. HNO_3_, CAUTION: potentially explosive) and a 2-methoxyethanol/HNO_3_ mixture (30 mL 2-methoxyethanol and 10 mL 2 M HNO_3_) as solvents. The samples were calcined using the protocol described above (pathway 1, Table S1 in Supporting Information File 1).

As mentioned above, bismuth is rather volatile at temperatures above 400 °C. Thus, an excess of bismuth is necessary to overcome bismuth loss during calcination. In order to study the influence of the bismuth nitrate excess with respect to iron nitrate, a series of reactions were performed using four different molar ratios between iron nitrate and bismuth nitrate, that is, 0% (equimolar ratio), 2%, 3%, and 5% excess of bismuth nitrate was used in the synthesis. The procedure was continued as described above using tartaric acid as complexing reagent and a 2-methoxyethanol/HNO_3_ mixture as solvent. The samples were calcined using the program described above (pathway 1, Table S1 in Supporting Information File 1).

To study the effect of different calcination procedures on the formation of undesired phases, the obtained powder was split into two parts and calcined at 500 °C for 1 h, using two different pathways: a) under the same conditions as described above (pathway 1) and b) the sample was inserted directly in the oven at 500 °C (see pathway 2, Table S1 in Supporting Information File 1). In both cases the samples were removed immediately after 1 h at 500 °C. The different syntheses are expressed as BiFeO_3_-#, *x*%, where # is the number of the calcination path and *x*% is the bismuth excess.

### Photocatalytic experiments

The photocatalytic activity of the samples was evaluated regarding the degradation of RhB in water at room temperature under visible light using high-power LEDs with an emission wavelength of λ > 420 nm as a light source. In a typical experiment, 50 mL of dye solution (concentration of rhodamine B = 5 mg/L) was mixed with 50 mg of the BiFeO_3_ sample. The mixture was stirred for 60 min in darkness to ensure an adsorption–desorption equilibrium between the catalyst and the dye solution. After irradiation, aliquots were taken every 30 min and centrifuged in darkness at 5000 rpm for 3 min to separate the catalyst powder from the solution. The absorbance of each sample during photocatalysis was measured at the maximum absorption peak of RhB.

### Characterization techniques and equipment

The structure and phase purity of the nanomaterials synthesized here were characterized using powder X-ray diffraction with a Bruker D2 Phaser diffractometer equipped with a Cu tube (λ = 1.54 Å) and a LYNXEYE XE-T detector. Crystalline impurities were identified using MATCH! Phase Identification from Powder Diffraction. Size and morphology of the samples were obtained using a FEI Tecnai G2 spirit twin transmission electron microscope (TEM). Fourier-transform infrared (FTIR) spectra of the nanoscale materials were recorded using a Jasco FT IR-4700 spectrometer. Bandgap information was obtained using the spectra recorded in a Perkin Elmer UV–vis spectrometer with an integrating sphere. Spectra were suitably transformed by a Kubelka–Munk model in order to obtain the bandgap energy value [43]. The absorption spectrum of RhB was measured using a UV–vis spectrophotometer GENESYS 30TM with tungsten-halogen light source and silicon photodiode detector. The spectra were fitted with the Thermo Scientific VISIONlite PC software suite.

## Results and Discussion

### Synthesis of BiFeO_3_ nanoparticles

**Figure 1:**
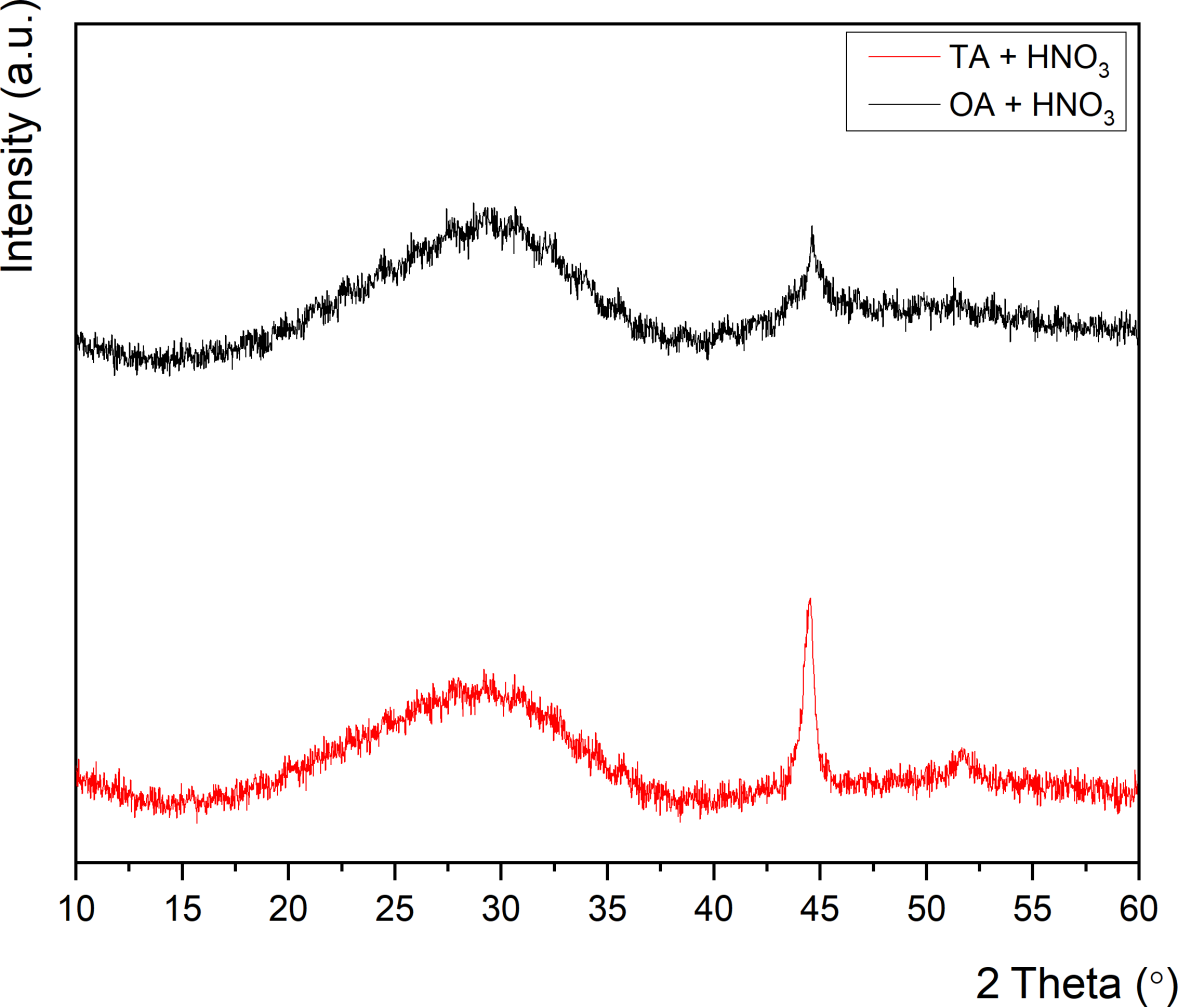
X-ray diffractograms when using different complexing agents.

In the first series of reactions we studied the impact of different solvents and chelating reagents on the synthesis of BiFeO_3_ using SBA-15 with a pore diameter of 5.5 nm as a hard template. When using oxalic acid (OA) or tartaric acid (TA) as complexing reagents and HNO_3_-acidified water as solvent, we found only amorphous products, as observed in the X-ray diffractograms in Figure 1.

**Figure 2:**
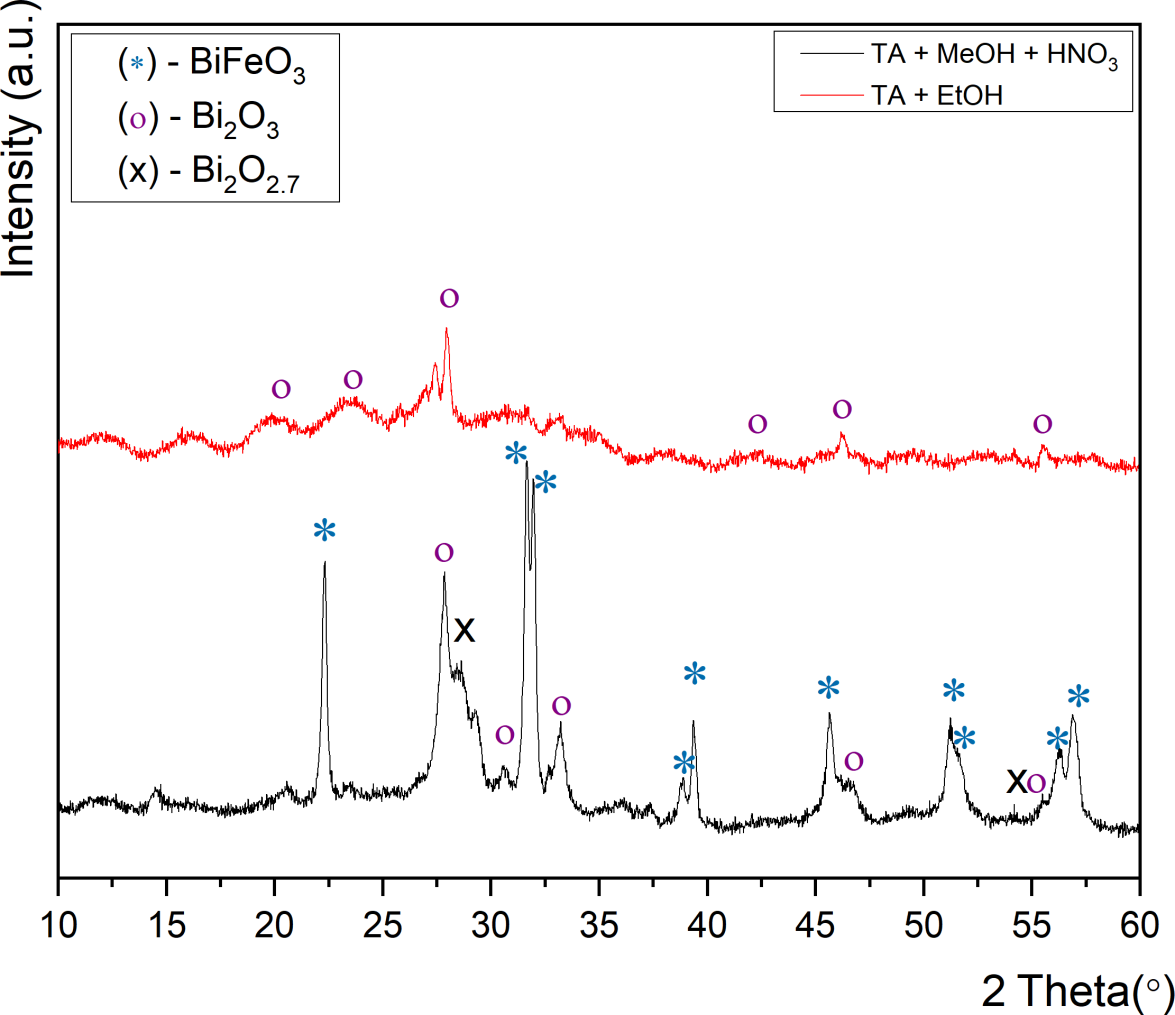
X-ray diffractograms when using different solvents.

This result can be attributed to the incorporation of Lewis-acidic metals, such as bismuth, into the amorphous silica framework during the reaction, which leads to overall amorphous products [44–47]. The impact on the phase formation of BiFeO_3_ of using different alcohols, that is, 2-methoxyethanol and ethanol as solvents and tartaric acid as a complexing reagent in the synthesis is shown in Figure 2.

Ethanol is commonly used in nanocasting syntheses. However, we have found that the metal precursors resulting from the reaction between the metal nitrates and tartaric acid are almost insoluble in neat ethanol [38,44,48]. It has been shown previously that both solvents can interact with metal nitrates resulting in the formation of metal–alkoxide complexes [49]. Since a clear solution is essential for the wet impregnation of SBA-15, we acidified carefully the ethanol-based reaction mixture with HNO_3_. The diffractograms of the resulting products show that slightly acidified 2-methoxyethanol (MetOH) as solvent produced better results regarding the formation of crystalline BiFeO_3_. Approximately 50.5% of the crystalline products seen in the diffraction patterns correspond to BiFeO_3_ (Figure 2).

**Figure 3:**
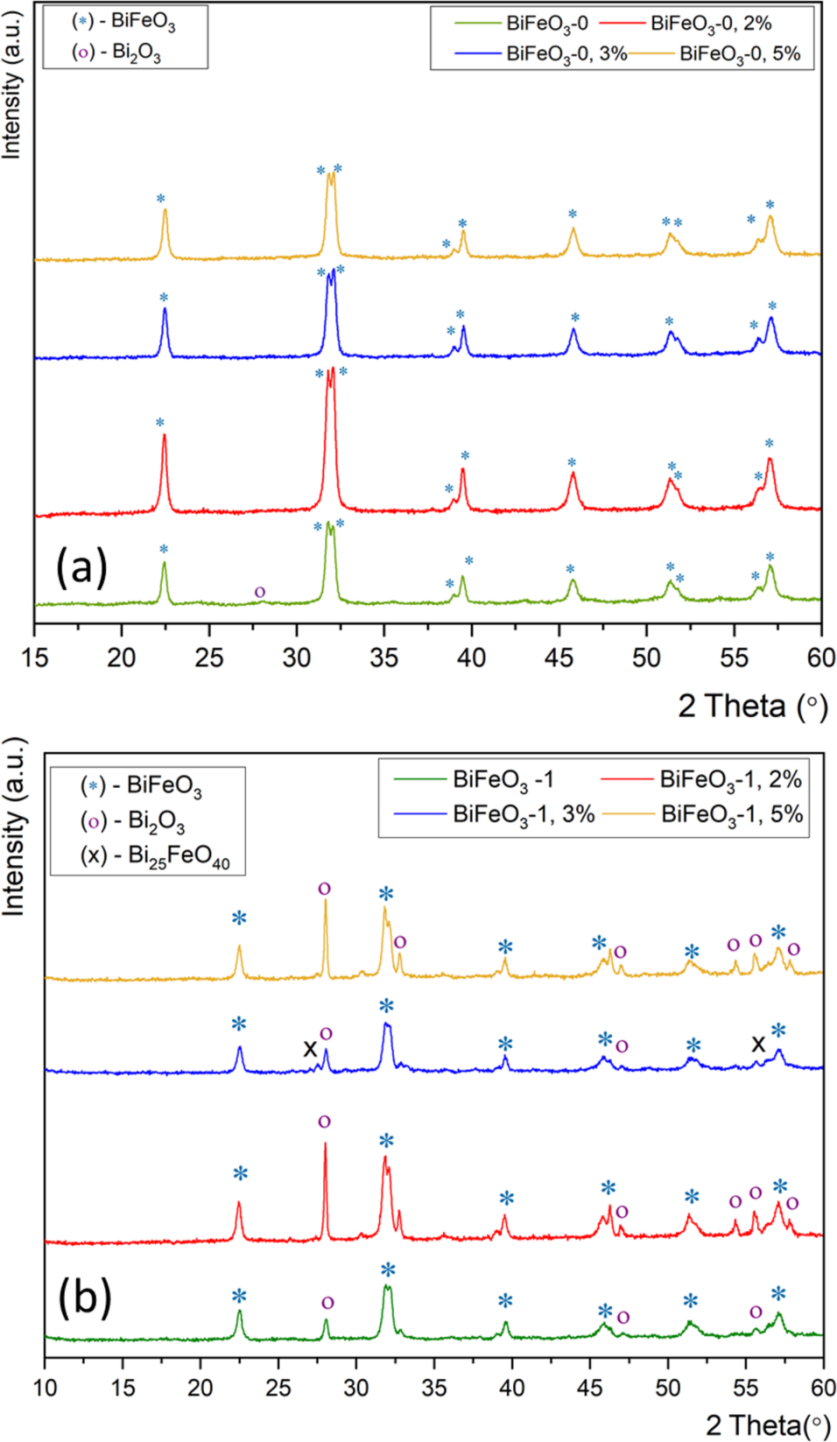
(a) X-ray diffractograms of BiFeO_3_-0, *x*% samples with equimolar, 2%, 3% and 5% of bismuth excess, calcined at intermediate temperatures. (b) X-ray diffractograms of BiFeO_3_-1, *x*% samples with equimolar, 2%, 3% and 5% of bismuth excess, without calcination at intermediate temperatures.

The peak splitting of the (104) and (110) peaks confirm the formation of a rhombohedral BiFeO_3_ perovskite structure (*R*3*c*) [19,33,50]. However, significant amounts of impurities could be identified in the samples, which can be assigned to Bi_2_O_3_ (approx. 33%) and Bi_2_O_2.7_ (16.5%). It should be noted that the Bi/Fe carboxylate intermediates resulting from the mixing of the corresponding metal nitrate precursors and tartaric acid are mostly insoluble in ethanol. Thus, acidification with HNO_3_ was very important in order to get homogeneous solutions (CAUTION: ethanol/HNO_3_ mixtures are potentially explosive). The use of acidified ethanol resulted primarily in the formation of Bi_2_O_3_. It should be noted that other solvent/ligand combinations such as methanol, dimethylformamide, acetonitrile, hexane/water mixtures, and toluene as well as toluene/water mixtures, in the presence or absence of tartaric acid and oxalic acid, lead either to the formation of multiple phases or amorphous products. In order to improve product formation using 2-methoxyethanol as a solvent, the effect of different molar ratios between the Bi/Fe metal precursors was then analyzed, since significant bismuth loss can be observed during calcination as bismuth is volatile at high temperatures. Thus, it is necessary to compensate for this loss during the heating step by adding excess bismuth. For this we carried out a systematic study using an equimolar ratio of Fe and Bi (labeled as BiFeO_3_-0) as well as 2%, 3% and 5% bismuth excess (BiFeO_3_-0, 2%, BiFeO_3_-0, 3% BiFeO_3_-0, 5%). Figure 3a shows the diffractograms for the products of using different molar Fe/Bi ratios.

All diffractograms show the formation of rhombohedral BiFeO_3_ as the main phase (space group *R*3*c*). For the equimolar reaction, the resulting diffractogram shows a minor impurity of around 2.8% at 2θ = 27.96°, which can be assigned to Bi_2_O_3_. The reactions using an excess of bismuth resulted in the formation of pure-phase rhombohedral BiFeO_3_. The diffractogram of BiFeO_3_-0, 5% shows similar results as BiFeO_3_-0, 3%. This means that the larger Bi excess is unnecessary to obtain the desired product. Thus, we decided to use a 3% bismuth excess for all further nanocasting reactions. It should be noted that due to the hygroscopic nature of bismuth nitrate, we observed higher yields and the formation of pure-phase BiFeO_3_ powders when fresh Bi(NO_3_)_3_·5H_2_O was used.

The influence of different calcination conditions was then analyzed. It should be noted that precursor powder resulting from the synthesis of bismuth and iron nitrates with equimolar amounts of tartaric acid in the presence of an alcohol can lead to an Fe^3+^-catalyzed autocombustion reaction. The rapid release of heat upon combustion can then lead to the formation of unwanted by-products [29,31]. Thus, the samples of BiFeO_3_-0, BiFeO_3_-0, 2%, BiFeO_3_-0, 3% and BiFeO_3_-0, 5% were split into two parts and a different calcination path was used for each part. Figure 3a shows the diffractograms of the samples that were calcined using intermediate plateaus at 200 °C for 2 h and then at 250 °C for 2 h.

Then, the samples were slowly heated to the final temperature of 500 °C at which they were calcined for another 1 h (pathway 1, Table S1 in Supporting Information File 1). The second set of samples, denoted as BiFeO_3_-1, BiFeO_3_-1, 2%, BiFeO_3_-1, 3% and BiFeO_3_-1, 5% was heated directly to the final calcination temperature without any intermediate plateaus (see Figure 3b for the corresponding X-ray diffractograms). A comparison of the diffractograms of all samples shows that the samples calcined without intermediate plateaus contained significant amounts of Bi_2_O_3_ and Bi_25_FeO_40_ impurities, whereas the samples calcined with intermediate platforms consisted of pure-phase BiFeO_3_ [29,51]. As described above, the usage of 2-methoxyethanol and metal nitrate precursors, as well as tartaric acid as a complexing reagent without intermediate prolonged drying steps could lead to a critical OH^−^/NO_3_^−^ ratio, which results in an Fe^3+^-catalyzed autocombustion process upon heating. In addition, the presence of Bi^3+^ in acidic aqueous solutions leads to the formation of polynuclear cationic species. Once a thermal treatment at 500 °C is applied, those clusters decompose into α- and β-Bi_2_O_3_ by-products, which are not observed when an intermediate heating step is carried out [29].

**Figure 4:**
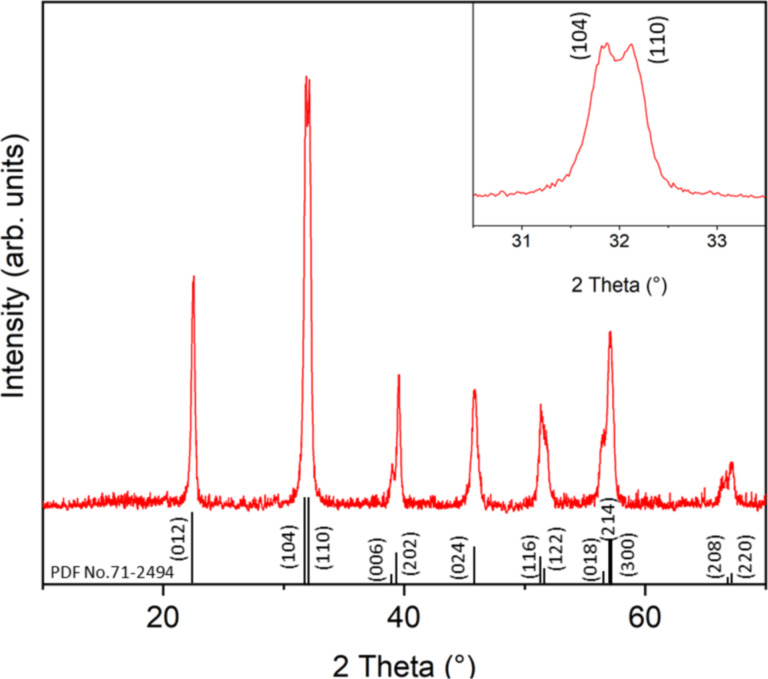
XRD pattern of 5.5 nm BiFeO_3_ NP.

Applying the findings regarding the influence of excess bismuth, type of complexing reagents, type of solvents, and calcination pathway, we then carried out the successful nanocasting of BiFeO_3_ nanoparticles. The TEM image of the impregnated and calcined BiFeO_3_@SBA-15 sample shows that the channels of the mesoporus silica host are still intact (Figure S6, Supporting Information File 1). Figure 4 shows the X-ray diffraction pattern at room temperature of the powdered BiFeO_3_ sample after the removal of the silica matrix.

**Figure 5:**
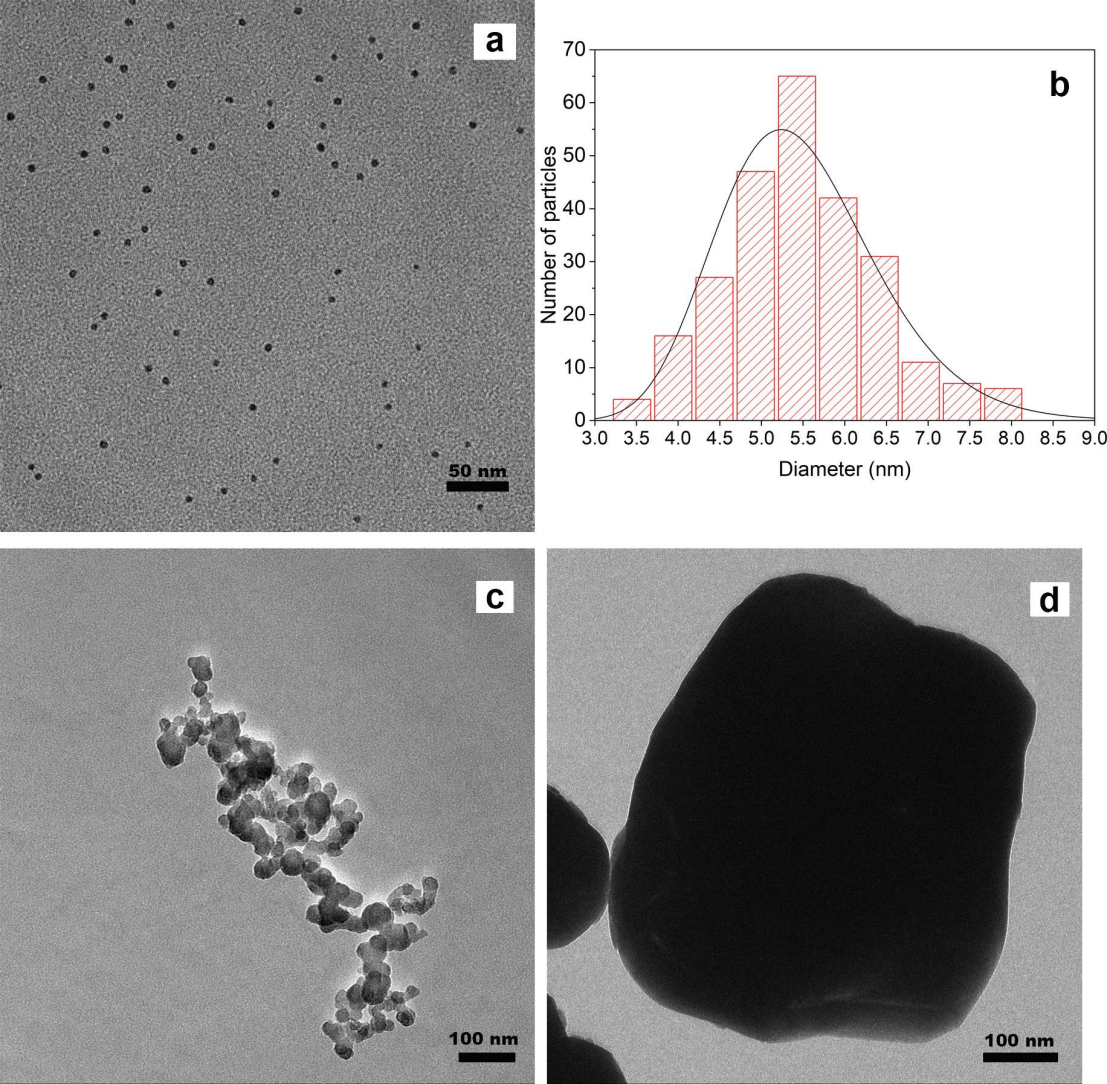
TEM of BiFeO_3_ samples (a) nanocast 5.5 nm BiFeO_3_ particles (b) particle size analysis (c) particles synthesized by a previously reported co-precipitation method, avg. size 20 nm (d) bulk material.

The XRD diffraction pattern indicates the formation of the pure-phase rhombohedral BiFeO_3_ perovskite structure (*R*3*c*) as evidenced by the peak splitting of the (104) and (110) peaks (see above). The formation of a rhombohedral structure is in agreement with BiFeO_3_ ceramics, while crystalline BiFeO_3_ films were shown to have a tetragonal structure [52]. TEM images (Figure 5a) show exclusively round particles with a particle diameter of 5.5 nm with a very narrow size distribution (standard deviation of ±1.0 nm on 258 analyzed particles, see histogram in Figure 5b). The monodisperse nature of the nanoparticles as well as their size of 5.5 nm confirms the successful wet impregnation of SBA-15 and the formation of BiFeO_3_ inside the pores of the host silica matrix. Since the sample was calcined at rather moderate temperature and with short calcination time, a further particle growth into wire-like structures was prevented and, consequently, the sample is characterized predominantly by non-agglomerated nanoparticles. We would like to point out that, to the best of our knowledge, the present pure-phase 5.5 nm BiFeO_3_ nanoparticles are the smallest nanoparticles of that material so far.

**Figure 6:**
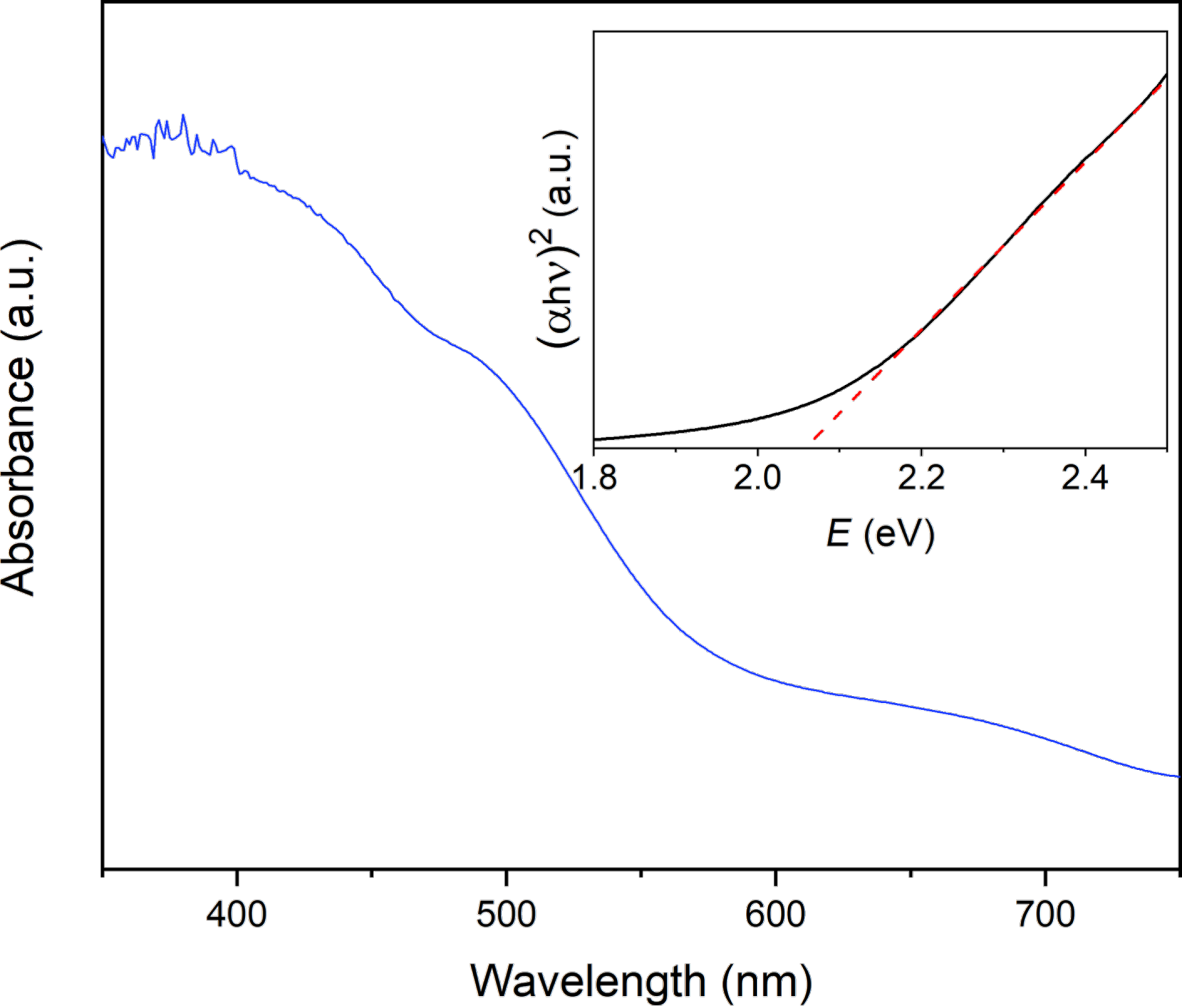
Absorbance spectra and Tauc plot (inset) for 5.5 nm BiFeO_3_ nanoparticles.

In order to investigate the optical properties of the nanocast BiFeO_3_ powder, diffuse reflectance UV–vis spectra were recorded and then transformed with the Kubelka–Munk method to obtain the bandgap energy. The analysis of the optical measurement reveals that the sample can absorb visible light over a wide range. The bandgap energy of the nanoparticles was then calculated from the tangent line in the plot of the square root of the Kubelka–Munk function vs the photon energy (Tauc plot, Figure 6).

The observed bandgap energy of 2.07 eV is considerably smaller than that of bulk BiFeO_3_ and comparable to those found in the literature for similarly sized particles [6,23,29,53]. However, we would like to point out that a comparison of bandgap values with different BiFeO_3_ samples is very difficult as particle shape, size, phase purity, as well as oxygen vacancies have a strong impact on the electronic structure of the resulting material.

### Photocatalytic activity

**Figure 7:**
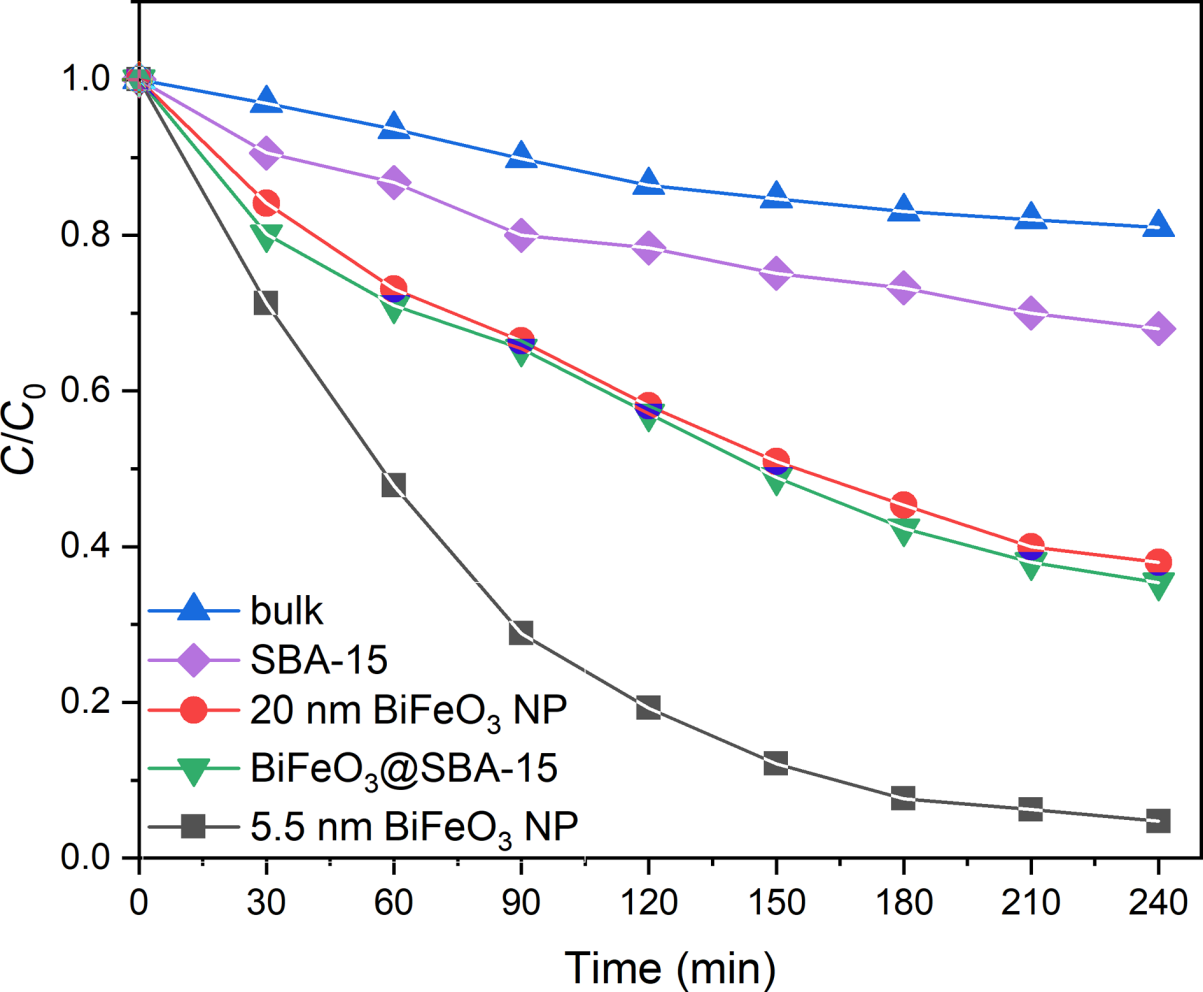
Photocatalytic degradation of RhB as a function of irradiation time under visible light using different catalysts.

The photocatalytic activity of all samples was evaluated using RhB as a model organic pollutant that is extremely stable under visible-light irradiation in the absence of a photocatalyst [28,51]. The photodegradation experiments clearly show that RhB has been degraded by the BiFeO_3_-0 5.5 nm catalyst as the intensity of the maximum absorbance peak of RhB at 553 nm decreased over time (see Supporting Information File 1, Figure S1). A closer inspection of that peak also reveals a blueshift of the maximum peak intensity from 553 to 548 nm due to the formation of intermediate species upon cleavage of the conjugated chromophore or removal of ethyl groups [54–55]. The degradation efficiency is evaluated by the ratio *C*/*C*_0_, in which *C*_0_ and *C* are the initial concentration of RhB and the RhB concentration as a function of the irradiation time, respectively. The nanocast nanoparticles degraded RhB by 95% after 240 min (Figure 7).

The catalytic performance in terms of overall degradation efficiency of the 5.5 nm particles is significantly superior compared to those of similarly sized particles reported. It also much improved when compared with samples free of surface defects synthesized by a previously reported co-precipitation method (Figure 5c) and bulk material (Figure 5d) [6]. The increase in efficiency when compared to larger particles can be explained by the lower bandgap and, certainly, by the higher surface area of the smaller particles. However, it was shown by Infante and Park that a reduction in particle size can actually lead to a decrease in photocatalytic activity due to crystal defects and local distortions altering the skin layer of the BiFeO_3_ photocatalysts [28,53,56]. From this results we conclude that the nanocasting method for the synthesis of BiFeO_3_ does not only produce pure-phase, uniform BiFeO_3_ with a very narrow particle size distribution. The results also suggest that the nanoparticles are characterized by a low concentration of surface defects and a low level of local strain, which is ideal for surface-based applications such as photocatalysis. This is confirmed by the reaction kinetics of the photodegradation of RhB. The rate constants of the photocatalytic degradations were calculated based on a Langmuir–Hinshelwood model (Equation 1):

[1] $$r=\frac{\text{d}c}{\text{d}t}=\frac{k_{r}K_{c}}{1+K_{c}},$$

with the reaction rate *r* (mg·L^−1^·min^−1^), reaction rate constant *k_r_* (mg·L^−1^·min^−1^), adsorption coefficient of the reactant *K_c_* (L·min^−1^), reactant concentration *c* (mg·L^−1^) and time of illumination *t* (min). For very small concentrations, as in the present case, Equation 1 can be converted to the following equation:

[2] $$r=-\frac{\text{d}c}{\text{d}t}=k_{r}K_{c}=kc,$$

where *k* (min^−1^) is the first-order rate constant. When *t* = 0, *C* = *C*_0_, Equation 2 can be simplified to

[3] $$\ln\left(\frac{C}{C_{0}}\right)=-kt.$$

**Figure 8:**
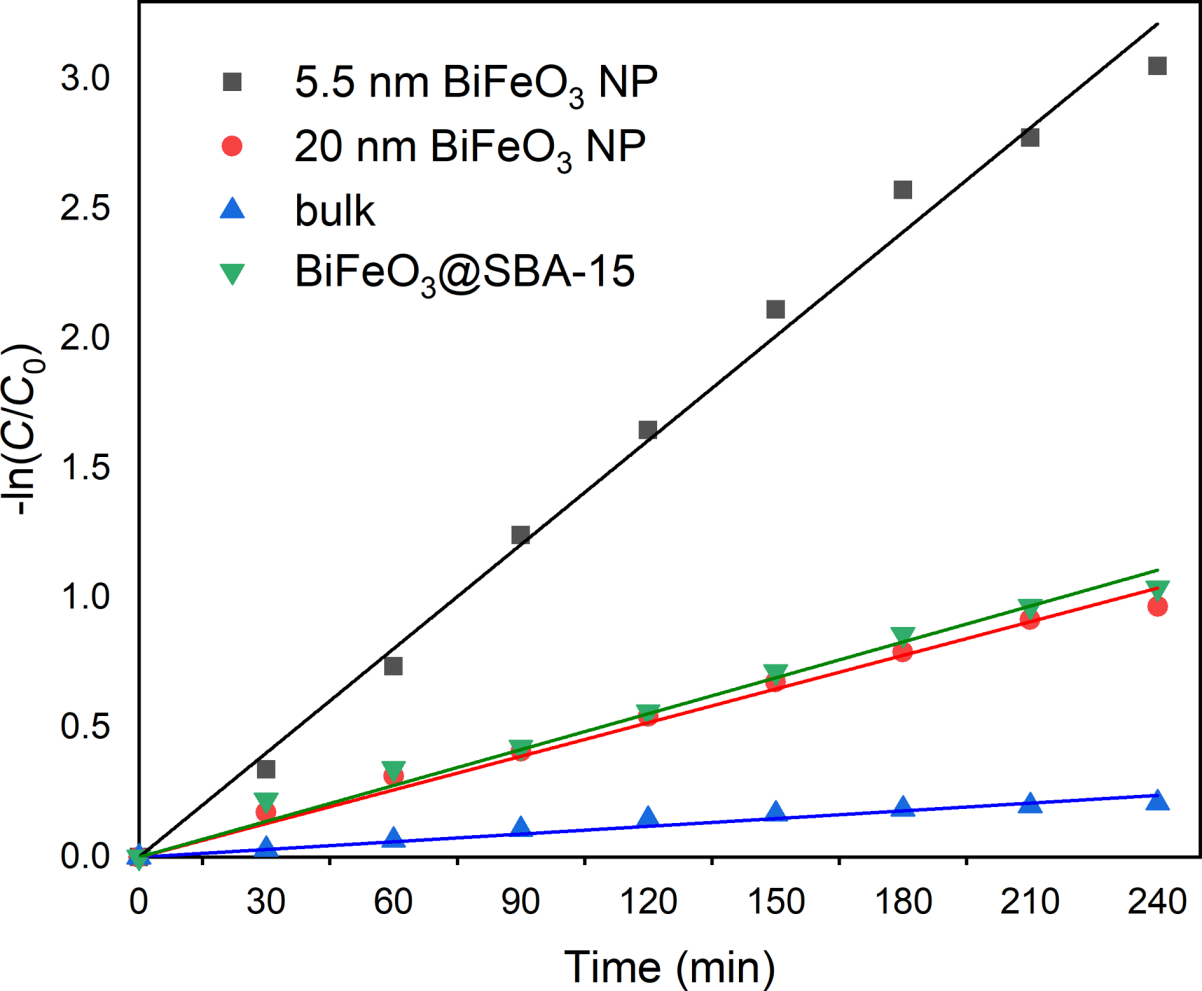
Kinetics of the photocatalytic degradation of RhB using different catalysts.

Thus, in accordance with Equation 3, ln(*C*/*C*_0_) as a function of the time *t* should follow a linear behavior with the reaction rate *k* being its slope. In agreement with the trends seen above, that is, smaller particles sizes resulting in better photocatalytical performance, the rate constant *k* increased starting from 9.99 × 10^−4^ min^−1^ (bulk BiFeO_3_) over 4.33 × 10^−3^ min^−1^ (BiFeO_3_, 20 nm) and 4.61 × 10^−3^ (BiFeO_3_ 5.5 nm@SBA, see below) to 1.34 × 10^−2^ (BiFeO_3_ 5.5 nm) and with decreasing particle sizes and thus increasing surface area (Figure 8).

The importance of the high surface area of the photocatalyst is also shown when the photodegradation of RhB is undertaken with BiFeO_3_ nanoparticles still being hosted in the silica matrix of SBA-15, that is, when the matrix has not been removed by NaOH leaching. Figure 6 shows that the degradation efficiency immediately drops by a factor of approximately 68% leading to an overall degradation of 64.7%. This is explained by the reduction of surface area available for the photodegradation of RhB since the catalyst particles are surrounded by the silica matrices. The reaction of mesoporous silica SBA-15 without the presence of a photocatalyst leads to an adsorption of the dye molecule by the silica matrix with a removal efficiency of 32%. It should be noted that all dye can be desorbed still undegraded from SBA-15 by a 2-methoxyethanol/H_2_O mixture [57–58].

**Figure 9:**
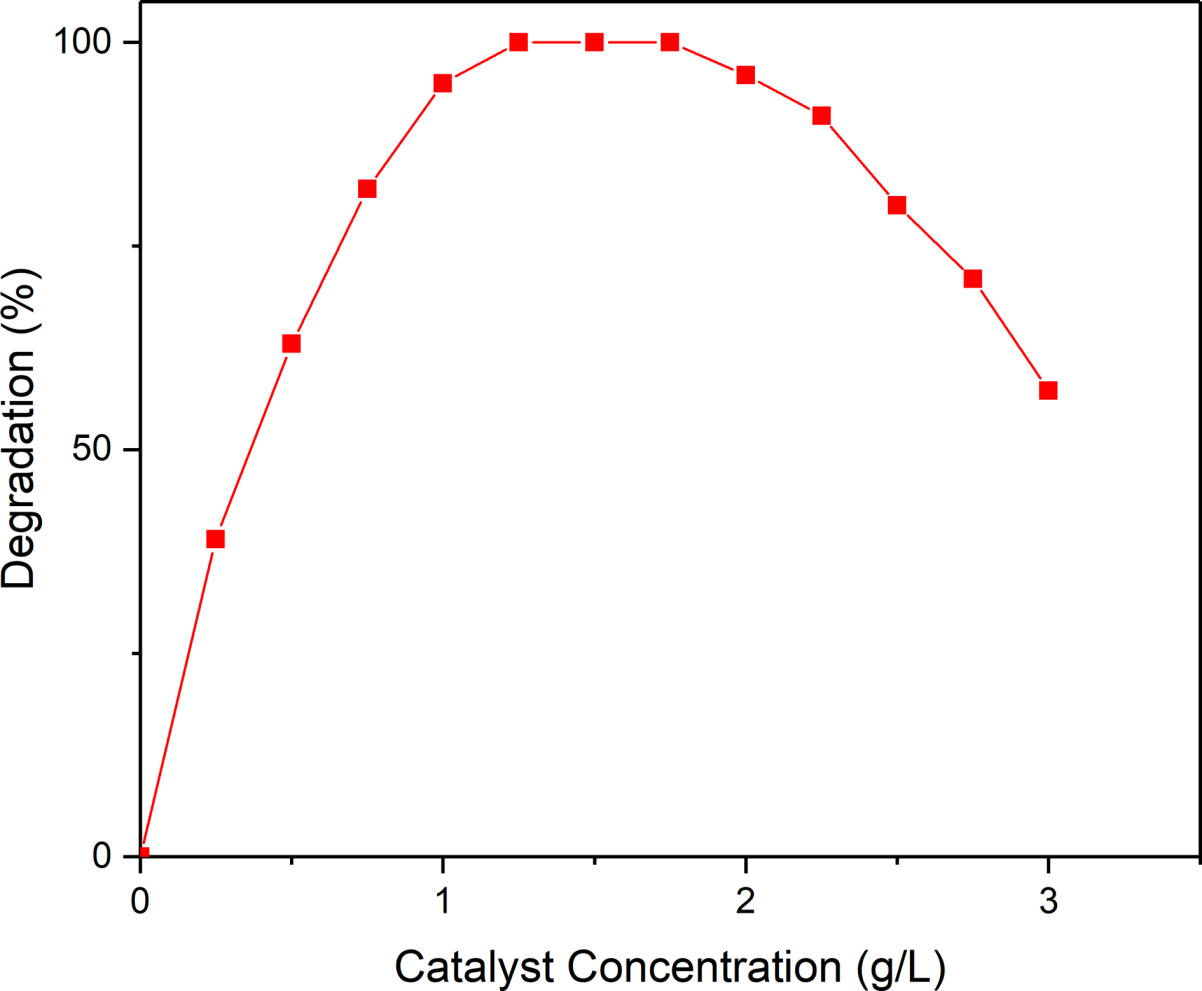
Effect of catalyst concentration on the degradation of RhB.

In order to study the effects of the catalyst concentration on the degradation reactions, a series of catalytic experiments were performed in which the concentration of the catalyst was varied from 0 to 3 g/L. As seen in Figure 9 the overall degradation of the dye at first increased until reaching a maximum of 100% at 1.25 g/L which can be explained by the increase in available active sites on the catalyst surface [15].

Catalyst concentrations greater than 1.75 g/L result in an increased opacity of the heterogeneous reaction mixture, which consequently leads to a decrease of the photocatalytic performance. Thus, we conclude that the optimum concentration of the nanocast BiFeO_3_ catalyst for the photodegradation of RhB is 1.25 g/L.

**Figure 10:**
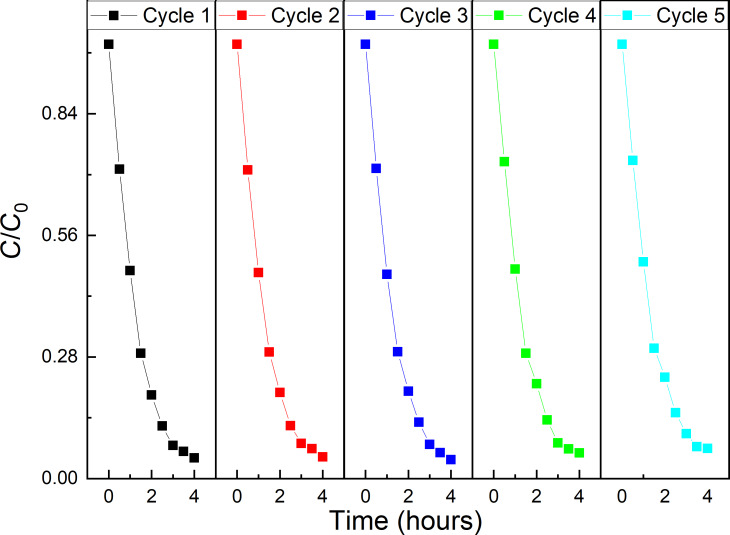
Evaluation of photocatalytic stability and reusability of 5.5 nm BiFeO_3_ NP in a total of five cycles.

The stability and reusability of the catalyst are very important factors in practical applications. Thus, the recyclability and photostability of the synthesized BiFeO_3_ nanoparticles was assessed in the degradation of RhB for five cycling runs (see Figure 10).

After each run, the catalyst was separated from the reaction mixture by means of centrifugation, washed once with distilled water, dried and reapplied in the next degradation experiment using a fresh RhB solution. After the last catalytic experiment, the catalyst was analyzed again by X-ray diffraction (see Figure S7 in the Supporting Information File 1). The results demonstrate that the catalyst retained its degradation efficiency without any significant loss of activity after five cycles, demonstrating the reusability and stability under the present conditions. The XRD diffraction pattern of the isolated catalyst confirms the stability of the catalyst since no crystalline impurity is detected.

**Figure 11:**
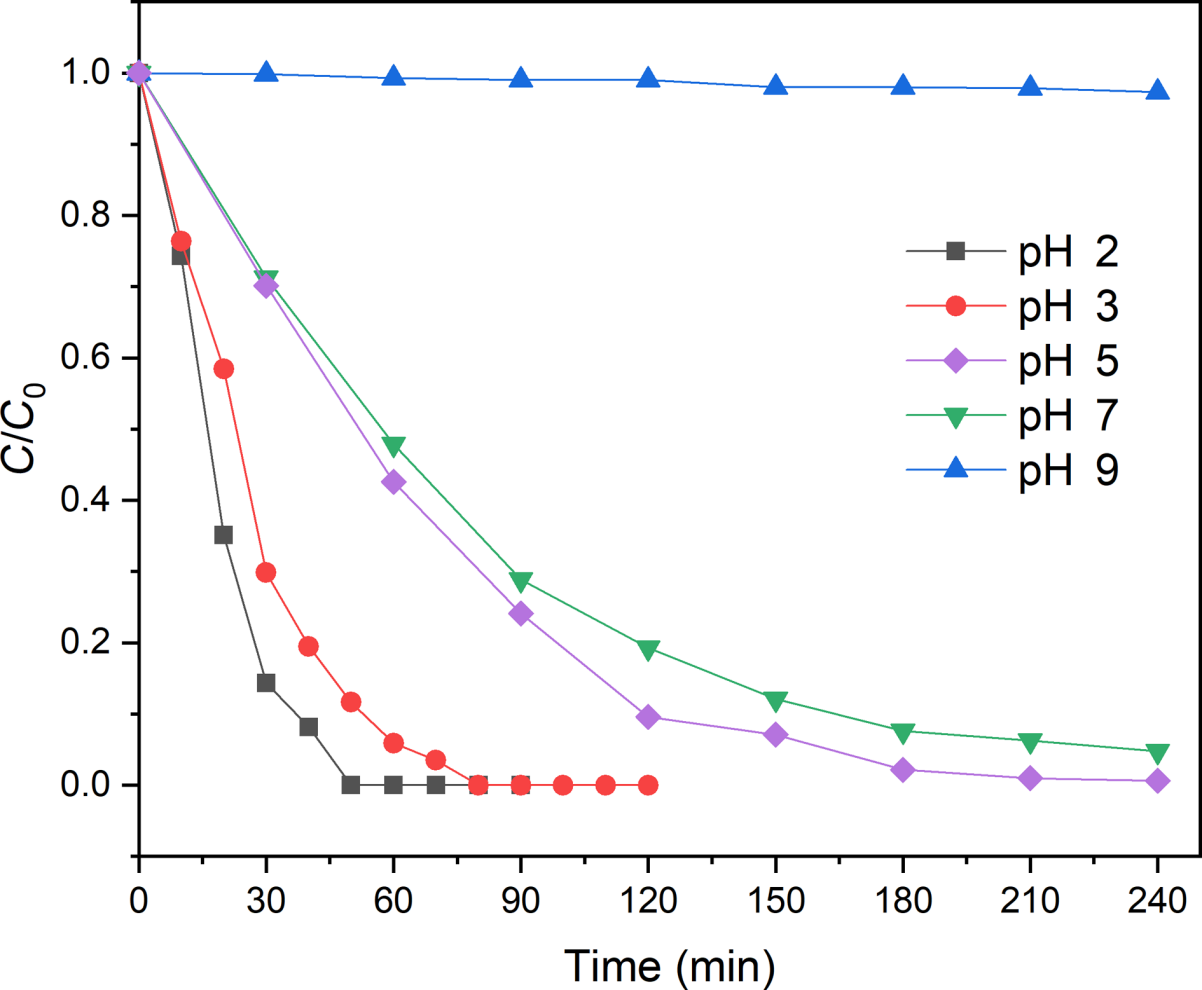
Photocatalytic degradation of RhB using 5.5 nm BiFeO_3_ NPs as photocatalyst under different pH condition.

The degradation efficiency of the 5.5 nm BiFeO_3_ particles in the presence of RhB at different pH values ranging from pH 2 to pH 9 was also investigated (Figure 11).

In general, varying the pH value of the reaction medium changes both structure and charge of the dye, as well as the surface-charge properties of the catalyst. For instance, at neutral pH values RhB is characterized by a zwitter ionic state, whereas it forms a cationic species upon protonation of a carboxylic group at pH values below its p*K*_S2_ value of 3.22. Under basic reaction conditions BiFeO_3_ catalyst surfaces are negatively charged, which leads to a repulsion of the negatively charged carboxylate functional groups of the dye molecule [51,54,59]. In addition, due to electrostatic interactions, RhB dimers are formed, which affects the charge density throughout the molecule and possibly leads to an interference in the degradation process [51]. In addition, the concentration of the active species, that is, hydroxyl radicals, decreases at pH > 7 due to their reaction with hydroxy anions and the formation of less oxidizing species such as O^−^. In accordance with the literature, the degradation efficiency increased with decreasing pH value. Remarkably, a complete degradation of RhB could be achieved within just 50 min by lowering the pH value to 2 as a consequence of the attractive interactions between the dye molecule and the catalyst surface upon protonation. In contrast, the photocatalytic activity almost ceased entirely under basic conditions at pH 9 due to the repulsive interactions of the formed carboxylate anions of the dye molecule with the catalyst surface (see above).

We then investigated the reaction mechanism by determining the active species. A series of trapping experiments with different scavengers, such as AgNO_3_, ethylenediaminetetraacetic acid (EDTA), *tert*-butyl alcohol (TBA), and benzoquinone (BQ), was performed. It is generally well-accepted that in semiconductor photocatalysis the reaction of promoted electrons with molecular oxygen leads to superoxide anion radicals (•O^2−^) and hydrogen peroxide (H_2_O_2_), while the photogenerated electron hole h^+^ reacts with H_2_O to form hydroxyl radicals (•OH). The latter species can additionally be formed by disproportionation of •O^2−^ radicals and a subsequent chain reaction. It has been reported previously that hydroxyl radicals are the main active species in photocatalytic degradation reactions [22].

**Figure 12:**
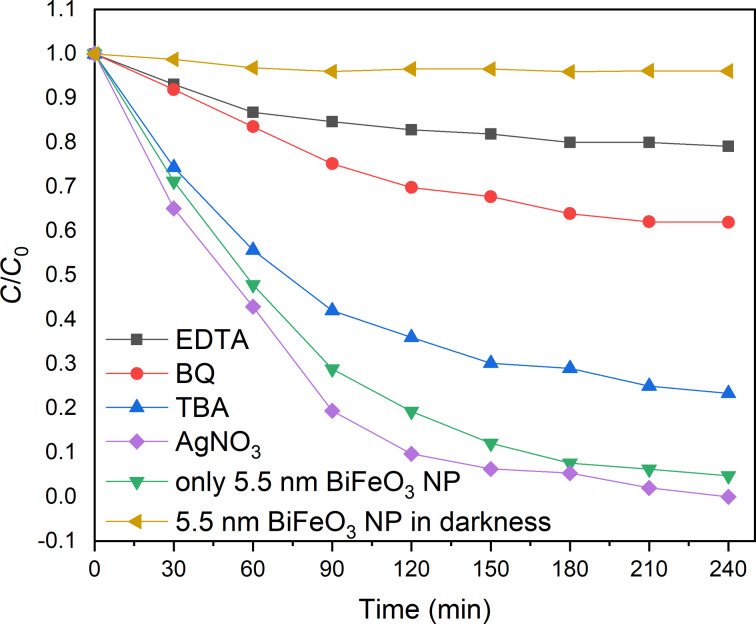
Mechanistic investigation of the photocatalytic degradation of RhB by using different radical scavengers.

The addition of silver nitrate (AgNO_3_, 2 mM) to the photocatalytic reaction leads to an improvement of the overall efficiency resulting in a complete degradation of RhB after 240 min (Figure 12).

This is explained by the consumption of excited electrons by the electron scavenger AgNO_3_ and the resulting enhanced separation of electron–hole pairs [60]. Photogenerated electron holes could play an important role in the present reaction, as the addition of the electron-hole scavenger EDTA (2 mM) decreases the degradation efficiency of the catalyst, leading to an overall degradation of approx. 21%. In order to evaluate the role of hydroxyl radicals, the radical scavenger *tert*-butyl alcohol (TBA, 2 mM) was added. The degradation percentage of RhB was reduced from 95% to 38% indicating that hydroxyl radicals play a major role in the photocatalytic oxidation process. At last, benzoquinone (BQ, 0.05 mM), which is a well-known superoxide radical scavenger was added resulting in a reduction of the degradation percentage from 95% to 76% [61]. In summary, the mechanistic study indicates that •OH radicals, photogenerated holes (h^+^) and superoxide radicals (•O^2−^) are all contributing to the degradation of RhB with the hydroxyl radical as the main active species. The photogenerated electrons are not taking part in the reaction (see Figure S2 in Supporting Information File 1).

## Conclusion

In summary, highly crystalline, monodisperse 5.5 ± 1 nm BiFeO_3_ nanoparticles were successfully synthesized by a nanocasting technique using SBA-15 as hard template. The structural analysis by XRD reveals pure-phase rhombohedral perovskite BiFeO_3_ with the space group *R*3*c*. In the synthesis, tartaric acid as complexing reagent and acidified 2-methoxyethanol as solvent, as well as 3% excess of Bi are essential to obtain pure-phase nanoparticles. Furthermore, calcination conditions show to be strongly correlated to the formation of this material. Here, intermediate plateaus and a final low calcination temperature of 500 °C proved to be necessary in order to avoid the formation of unwanted phases such as Bi_2_O_3_ and Bi_25_FeO_40_. The synthesized nanoparticles were used as a photocatalyst in the photodegradation of RhB under visible-light irradiation. The nanoparticles are characterized by an enhanced photocatalytic activity when compared to similarly sized particles. This behavior can be attributed to a low concentration of surface defects and low local strain. The photocatalyst proved to be stable under visible-light irradiation as it could be reapplied successfully in five successive photocatalytic degradation experiments without loss of degradation efficiency. Under optimized reaction conditions, the complete degradation of the organic dye molecule could be achieved in less than 50 min. Kinetic studies show that the photodegradation follows first-order kinetics based on the Langmuir–Hinshelwood model. The main active species in the degradation are hydroxyl radicals (•OH), photogenerated holes (h^+^) and superoxide radicals (•O^2−^) as mechanistic studies showed.

## Supporting Information

File 1 Additional figures and tables.
