# Supplementary material for: Nanocasting synthesis of BiFeO3 nanoparticles with enhanced visible-light photocatalytic activity
Source: Beilstein J Nanotechnol. 2020 Dec 7;11:1822–33. doi: 10.3762/bjnano.11.164 (PMC7736686; doi:10.3762/bjnano.11.164)
Supplement: File 1 — Additional figures and tables. [file Beilstein_J_Nanotechnol-11-1822-s001.pdf]

BEILSTEIN JOURNAL OF NANOTECHNOLOGY

Supporting Information

for

Nanocasting synthesis of $BiFeO_3$ nanoparticles with enhanced visible-light photocatalytic activity

Thomas Cadenbach, Maria J. Benitez, A. Lucia Morales, Cesar Costa Vera, Luis Lascano, Francisco Quiroz, Alexis Debut and Karla Vizuete

Beilstein J. Nanotechnol. **2020,** *11,* 1822–1833. doi:10.3762/bjnano.11.164

Additional figures and tables

License and Terms: This is a supporting information file under the terms of the Creative Commons Attribution License (https://creativecommons.org/licenses/by/4.0). Please note that the reuse, redistribution and reproduction in particular requires that the author(s) and source are credited and that individual graphics may be subject to special legal provisions.
The license is subject to the *Beilstein Journal of Nanotechnology* terms and conditions: (https://www.beilstein-journals.org/bjnano/terms)

Table S1: Calcination paths.

Path	Reaction regarding: complexing agents, solvents, molar ratio-0	Reaction regarding molar ratio-1
**Initial temperature (°C)**	22	-
**Heat rate 1 (°C/min)**	1	-
**Plateau 1 (°C)**	200	-
**Drying Time 1 (h)**	2	-
**Heat rate 2 (°C/min)**	1	-
**Plateau 2 (°C)**	250	-
**Drying Time 2 (h)**	2	-
**Heat rate 3 (°C/min)**	4	4
**Final Temperature (°C)**	500	500
**Final Calcination Time (h)**	1	1

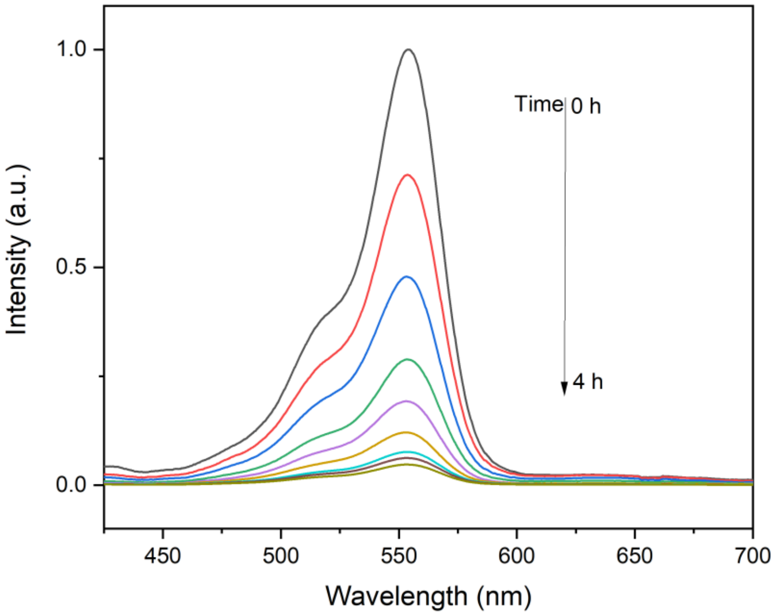

Figure S1: UV–vis spectra of Rhodamine B in dependence of irradiation time in the presence of 5.5 nm $BiFeO_3$ NP.

$$BiFeO_3 + h\nu \rightarrow e^- + h^+$$
$$h^+ + H_2O \rightarrow \cdot OH$$
$$h^+ + RhB \rightarrow \text{degradation products}$$
$$\cdot OH + RhB \rightarrow \text{degradation products}$$
$$\cdot O^{2-} + RhB \rightarrow \text{degradation products}$$

Figure S2: Photocatalytic mechanism of RhB degradation using 5.5 nm $BiFeO_3$ nanoparticles.

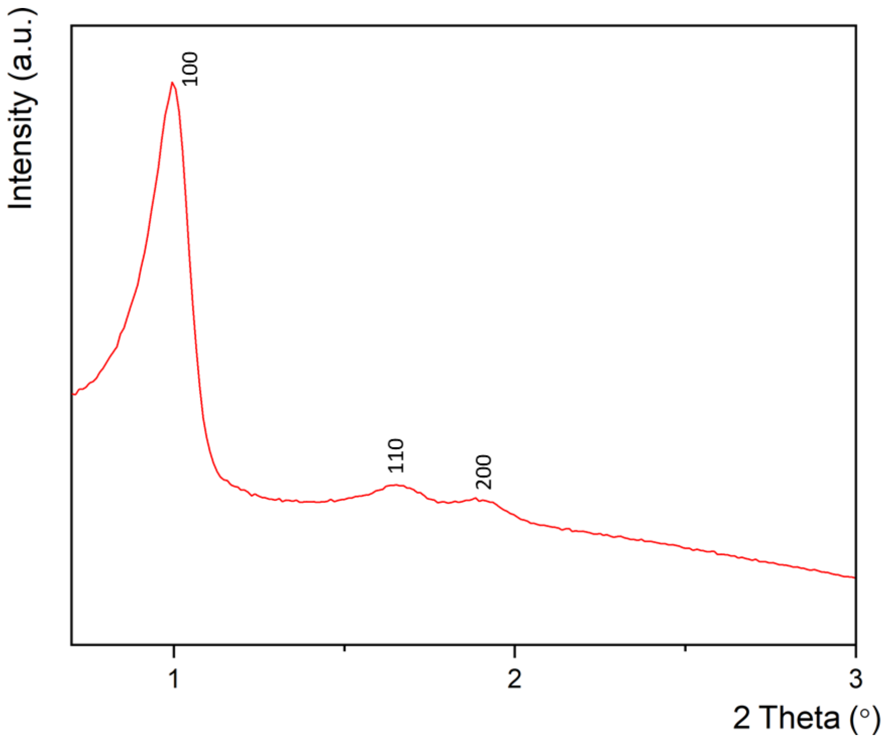

Figure S3: Small-angle XRD pattern of SBA-15.

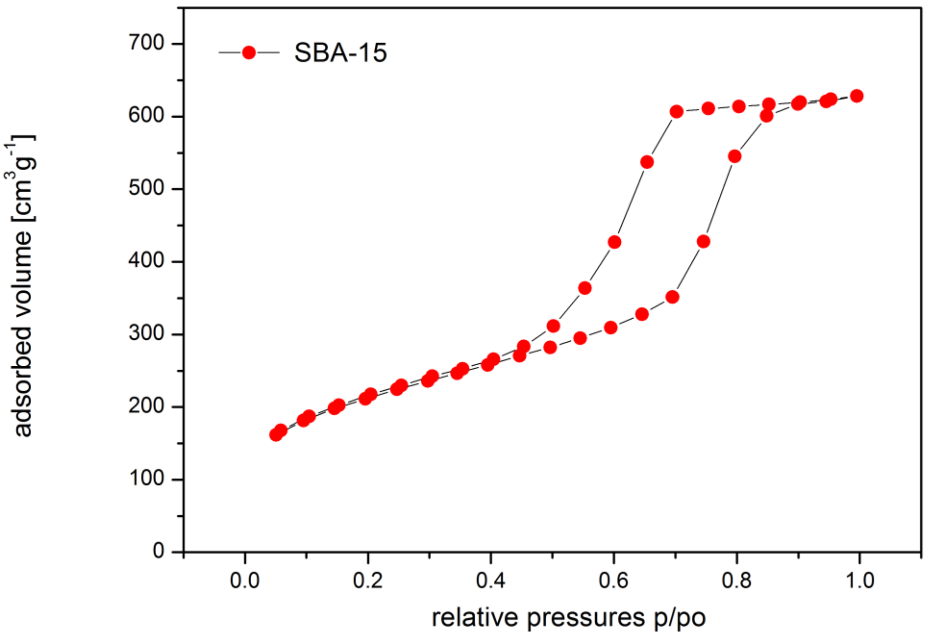

Figure S4: Nitrogen adsorption isotherms of SBA-15.

Table S2: Summary of BET analysis of SBA-15.

**Pore Size**	
Adsorption average pore diameter (4V/A by BET):	6.01773 nm
Desorption average pore diameter (4V/A by BET):	6.05495 nm
BJH Adsorption average pore diameter (4V/A):	6.2516 nm
BJH Desorption average pore diameter (4V/A):	5.8164 nm
D-H Adsorption average pore diameter (4V/A):	6.2643 nm
D-H Desorption average pore diameter (4V/A):	5.8198 nm

**Surface Area**	
Single point surface area at P/P_o = 0,300000000:	680.2137 m^2/g
BET Surface Area:	699.0213 m^2/g
Langmuir Surface Area:	3,573.7975 m^2/g
t-Plot Micropore Area:	33.0353 m^2/g
t-Plot external surface area:	665.9860 m^2/g
BJH Adsorption cumulative surface area of pores	
between 1.7000 nm and 300.0000 nm diameter:	651.433 m^2/g

BJH Desorption cumulative surface area of pores	
between 1.7000 nm and 300.0000 nm diameter:	716.5886 m^2/g
D-H Adsorption cumulative surface area of pores	
between 1.7000 nm and 300.0000 nm diameter:	647.872 m^2/g
D-H Desorption cumulative surface area of pores	
between 1.7000 nm and 300.0000 nm diameter:	714.5655 m^2/g

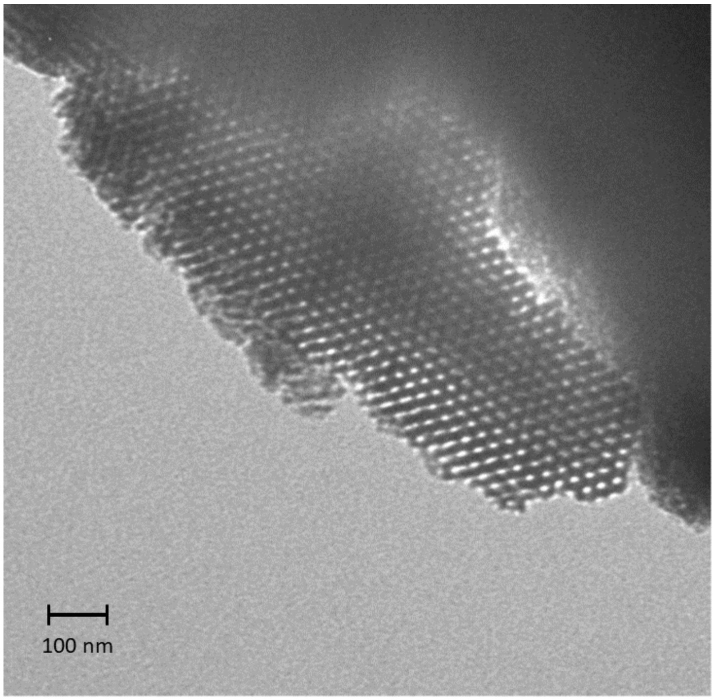

Figure S5: TEM image of SBA-15.

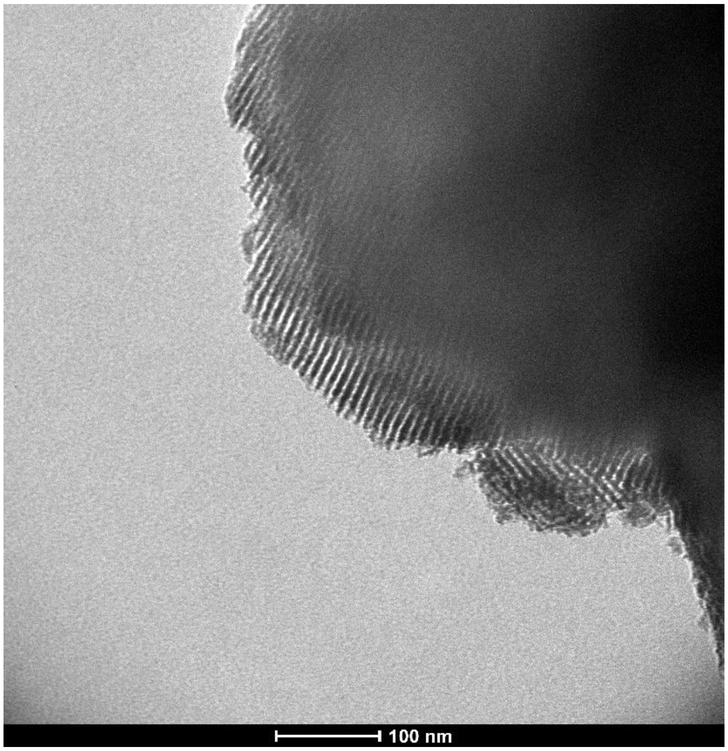

Figure S6: TEM image of $BiFeO_3$@SBA-15.

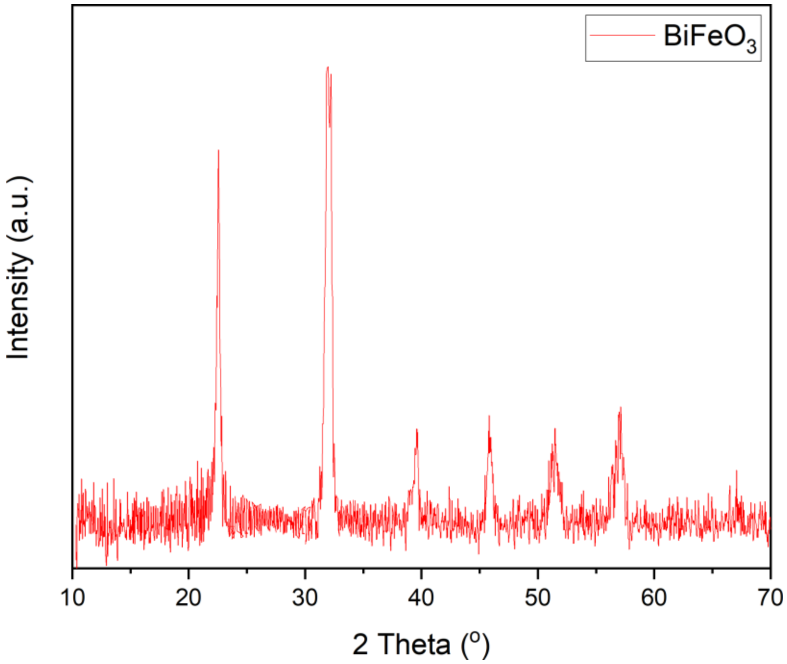

Figure S7: XRD pattern of 5.5 nm $BiFeO_3$ NP after 5 catalytic cycles.
